# Global funding for local health issues

**DOI:** 10.2471/BLT.15.030615

**Published:** 2015-06-01

## Abstract

Jeremy Farrar tells Fiona Fleck why global health research must go local to respond to social needs.

Q: The Wellcome Trust was set up in 1936 and has been a major player in many scientific advances, including sequencing the human genome. It’s the world’s second largest health research funder after the Bill & Melinda Gates Foundation with an endowment of £19 billion (US$ 29.5 billion). How did it feel to take over the reins as director of the Wellcome Trust in October 2013?

A: Tremendous excitement but of course also daunted by the responsibility. The first outweighs the second, and you grasp the amazing opportunity and seek to contribute what you can. It is an enormous privilege and honour to be the director of such an organization. Having been funded by the Wellcome Trust as a researcher for almost two decades, I knew it very well from the outside. I knew its approach to research, its history and ethos and its values and many of the people working there. This was incredibly helpful when I took over as director.

Q: To what extent are you funding individual researchers as opposed to collaborations?

A: Globally there has been a trend over the past 20 years – and this has been true of almost every major funder – to increasingly focus both on individuals and very large consortiums of researchers. There are stellar individual researchers out there and I strongly believe in funding the best people, but I also believe in funding the best people working in great teams in great environments. I also believe in funding young people. Writing a book is an individual pursuit and there are areas of science where a brilliant individual working alone makes a breakthrough. But the world of research is increasingly about people working in multidisciplinary teams that consider the social context of their work and are not working in a scientific vacuum. While I believe we must continue to support individual researchers to explore their dreams, there is also a need to put back into the heart of research funding the opportunity for groups of researchers to work together and integrate the broader social consequences of their work. That is why we recently introduced collaborative grants to encourage teams of researchers to work together within or between disciplines.

Q: What other new initiatives have you brought in?

A: Major scientific breakthroughs are often made by relatively young people in their 20s or 30s. I felt we need to back young people taking on what might seem improbable ideas and encourage them. There has been a tendency, and not just at the Wellcome Trust, to move away from funding young people and award very large grants to people with long established track records. We should support well-established scientists, but we risk disenfranchising young people. We need to support the development of the next generation of research leaders.

Q: What research funding model would you like to see and what are you doing to support this?

A: We need a mixed model which supports individuals throughout their careers, encourages people to take risks and, when driven by the research, allows people to work in teams which take a broad view of the question, including the social context of the issues. Recently we took steps to increase the opportunities for early and mid-career researchers. We also introduced seed grants to allow people who take on risky projects to gain initial data and apply for larger funds to further develop their projects.

Q: The Trust has been criticized in the past for a lack of transparency over the reasons for specific grant awards. How are you making the decision-making process more transparent?

A: It is fair to say that we have not always provided as detailed and constructive feedback as we might have done. We get a huge number of applications and we cannot fund everyone. We are aware of the importance of feedback and aim to make more effort to provide feedback to unsuccessful applicants on the reasons why their applications were not funded and how applications can be improved. This feedback is particularly important for young researchers.

Q: How do you decide who to fund?

A: In the Wellcome Trust we stimulate ideas, advise and encourage research grant applicants. But most of the decisions on which grants receive funding are made by the committees of external experts that peer-review applications and interview candidates. We are present at the committee meetings to ensure that the process is fair and that the committees’ work is aligned with the Trust’s strategic areas. The Trust decides the level of funding for each of our five strategic directions and each grant’s funding comes out of the funds that are flexibly allocated for the relevant strategic areas.

Q: How flexible is the Trust’s funding if the committees make the decisions?

A: We are not fixed in our allocation of funding and are able to move funding between our strategic directions to support the most promising research – research that we hope will have the greatest impact.

Q: Is there a shift away from awarding grants to recipients from the United Kingdom and Commonwealth countries?

A: The Commonwealth doesn’t come into it, we are a global organization. We fund research in some Commonwealth countries, but also in many others, such as China, Indonesia, Lao People's Democratic Republic, Nepal, Viet Nam and others. Under my predecessor Mark Walport, the Trust’s spending on grants outside the United Kingdom increased from approximately 11–12% to 23–25% and I hope we can increase this even further. This is important to the Trust’s history and goes back to our founder, Henry Wellcome, who was an internationalist. We are a global funder of research with a focus on support for research that can make a difference to people’s lives. The United Kingdom has a very strong basic science, clinical and humanities research base, but the Trust is committed to global health and is a global organization.

“We are a global funder of research with a focus on support for research that can make a difference to people’s lives.”

Q: Can you give some examples of projects that aim to do this?

A: We are shifting some decision-making and other activities to where the needs are. For example, we have set up a research hub with the Indian government, the Wellcome DBT India Alliance, which peer-reviews applications from Indian researchers, interviews candidates and decides which applicants should receive funding. This way research is defined at local level and not from an office in London. We have also recently created a research hub in Africa called the Alliance for Accelerating Excellence in Science in Africa. This is a partnership with the United Kingdom’s Department for International Development, the Bill & Melinda Gates Foundation and we hope others in future. The African programme is now transitioning from London to the African Academy of Sciences in Nairobi, where the peer-review process and interviews for grants will be led by the African Academy of Sciences. These two major initiatives support the development of the next generation of research leaders based in Africa and India. I hope to work towards further initiatives in the future in eastern Asia and other parts of the world.

Q: One of the issues you have taken up is antimicrobial resistance, why?

A: Antimicrobial resistance is the most important emerging infectious disease problem of our time. Currently we are all working in siloes. We need to bring together people working in different disciplines: the human and animal health sector, people working in economics, social sciences, anthropology, law and policy to respond to this massive and growing problem. The whole of modern medicine relies on having effective antibiotics to prevent and treat infections. Without antibiotics basic surgery, chemotherapy for cancer and care of patients with diabetes, for example, become very difficult if not impossible. Addressing antimicrobial resistance is a major priority for the Wellcome Trust.

Q: What is the Trust doing to address the problem?

A: We are working closely with WHO, governments and philanthropic organizations around the world to make sure the issue of antimicrobial resistance is a priority so that collectively we act to reduce the burden of resistant infections, prevent new resistant strains emerging and develop new drugs (and use existing drugs better) and other interventions, for example, vaccination and sanitation, to treat resistant infections when they arise or – even better – to prevent them.

“Antimicrobial resistance is the most important emerging infectious disease problem of our time.”

Q: What have health funders learned from the current Ebola outbreak?

A: There are many lessons not just for funders but for everyone. Many of them are the same lessons from the severe acute respiratory syndrome (SARS) and H5N1 [avian influenza] outbreaks as well as from the development of artemisinin-resistant malaria, the current epidemic of MERS-CoV [Middle East respiratory syndrome coronavirus] and many other national and regional epidemics over the last decade. We need to be much better prepared to identify and report such infections when they emerge as well as other potential public health threats. We also need to be able to respond to these threats in a much more decisive and coordinated manner. Such a response requires much stronger public health and clinical systems in all countries but in particular low- and middle-income countries. It also requires honest, transparent and accountable reporting and sharing of data, equitable sharing of the benefits of that data (including affordable and real-time access for diagnostics, drugs, vaccines or social interventions) and much greater incentives for countries to work together and share information.

Q: How?

A: I hope that all of us – communities, funders, public health, clinical, governments, global organizations – can work together to really learn the tough lessons of the Ebola epidemic and put in place the systems and approaches that we increasingly need so that we can prepare for – and respond to – epidemics and other public health crises. With changing environments, habitats, urbanization, increased travel and changes in societies around the world we are inevitably going to face more national, regional and global epidemics.

Q: Where does research come in?

A: To mount the required response to future epidemics and health threats we need a strong research component that works in the inter-epidemic times to ensure that we have interventions that might be needed in times of crisis. During epidemics, we need a robust and ethical research framework to ensure that the information needed to guide the public health and clinical response can be acquired, shared and acted upon in real time. Importantly, we need to ensure that the knowledge, interventions and systems needed to prevent future epidemics and bring them under control are equitably shared among all those who need them.

